# Molecular Dynamics Studies on the Structural Characteristics for the Stability Prediction of SARS-CoV-2

**DOI:** 10.3390/ijms22168714

**Published:** 2021-08-13

**Authors:** Kwang-Eun Choi, Jeong-Min Kim, JeeEun Rhee, Ae Kyung Park, Eun-Jin Kim, Nam Sook Kang

**Affiliations:** 1 Graduate School of New Drug Discovery and Development, Chungnam National University, 99 Daehak-ro, Yuseong-gu, Daejeon 34134, Korea; hwendiv@naver.com; 2 Division of Emerging Infectious Diseases, Bureau of Infectious Disease Diagnosis Control, Korea Disease Control and Prevention Agency, 187 Osongsaengmyeong 2-ro, Osong-eup, Heungdeok-gu, Cheongju-si 28159, Korea; jmkim97@korea.kr (J.-M.K.); jerhee001@korea.kr (J.R.); parkak1003@korea.kr (A.K.P.); ekim@korea.kr (E.-J.K.)

**Keywords:** SARS-CoV-2, spike protein, mutant, MD simulation

## Abstract

Severe acute respiratory syndrome coronavirus 2 (SARS-CoV-2) affects the COVID-19 pandemic in the world. The spike protein of the various proteins encoded in SARS-CoV-2 binds to human ACE2, fuses, and enters human cells in the respiratory system. Spike protein, however, is highly variable, and many variants were identified continuously. In this study, Korean mutants for spike protein (D614G and D614A-C terminal domain, L455F and F456L-RBD, and Q787H-S2 domain) were investigated in patients. Because RBD in spike protein is related to direct interaction with ACE2, almost all researches were focused on the RBD region or ACE2-free whole domain region. The 3D structure for spike protein complexed with ACE2 was recently released. The stability analysis through RBD distance among each spike protein chain and the binding free energy calculation between spike protein and ACE2 were performed using MD simulation depending on mutant types in 1-, 2-, and 3-open-complex forms. D614G mutant of CT2 domain, showing to be the most prevalent in the global pandemic, showed higher stability in all open-complex forms than the wild type and other mutants. We hope this study will provide an insight into the importance of conformational fluctuation in the whole domain, although RBD is involved in the direct interaction with ACE2.

## 1. Introduction

Severe acute respiratory syndrome coronavirus 2 (SARS-CoV-2), an RNA virus of the family Coronaviridae, emerged in Wuhan, China, in 2019, as the cause of the coronavirus disease 2019 (COVID-19) pandemic [1]. In general, coronaviruses (CoVs) contain 16 nonstructural and 4 structural proteins. The four structural proteins include spike (S) glycoproteins and envelope (E), membrane (M), and nucleocapsid (N) proteins [2]. The envelope-anchored S protein is an important determinant of the specificity of viral interactions with the host because it causes the viral particles to protrude; it also makes the first contact with the host cell. The S protein is cleaved by the host protease to produce the N-terminus S1 subunit and C-terminus S2 subunit [3]. The molecular recognition and attachment of the CoV particles to the host cell arise from the binding of the receptor-binding domain (RBD) on the S1 subunit of the S protein to human angiotensin-converting enzyme 2 (ACE2) [4,5], transmembrane serine protease 2, the protein cleavage enzyme furin, and the extracellular matrix metalloproteinase inducer CD 147 [6]. The S1 subunit is comprised of the N-terminal domain, RBD, and C-terminal (CT) domains 0, 1, and 2. Based on the RBD position, the S protein structure takes one of two conformational states, closed or open [7]. The three RBDs are down in the closed state, whereas one, two, or all three RBDs are up in the open state. The transition of S protein from closed to open form provides accessibility to ACE2 binding. A maximum of three ACE2 molecules can bind to the RBDs with a full three open form conformation [8]. The binding mechanisms for ACE2 and RBD, which instigates antigenicity, have been the subject of active study in different conditions, such as temperature, environment, or species [9,10,11].

The 3D structures of almost half of the 26 SARS-CoV-2 proteins have been determined using X-ray crystallography and cryogenic electron microscopy techniques, some of them within weeks of the start of the pandemic. So far, more than 900 structures of SARS-CoV-2 proteins have been deposited in the Protein Data Bank (PDB) (www.rcsb.org) (accessed on 3 March 2021) for immediate use by the scientific community. The proteins with the most structures deposited are two 3C-like proteases of the virus, with more than 250 structures, and the S protein, with more than 230 structures. Computational approaches based on 3D structures have been used to develop a multitude of tools for forecasting the antigenic evolution of viruses, and these approaches are useful for research into the mechanisms of activity of proteins produced by the virus. Rath et al. analyzed the conformational sensitivity of the S protein to temperature differences [9]. Deganutti et al. calculated its binding affinity to small molecules using the RBD–ACE2 complex structure [10]. Nelson et al. simulated RBD for long times at the millisecond scale to confirm the flexible loop of RBD interacting with ACE2 in pre-fusion conformation [12]. Malaspina and colleagues used the structure of the whole S protein to analyze its interactions with the surfaces of other molecules [13]. To compare the RBD of the SARS-CoV-2 with that of SARS-CoV-1, which caused a previous outbreak, Spinello et al. [14] simulated and He et al. used a protein–protein interaction docking method through the RBD–ACE2 interaction [15]. Peng carried out the free energy calculation in the system to identify differences in binding with ACE2 for SARS-CoV-2 and SARS-CoV-1 [16]. Wang et al. calculated the hydrogen-bonding network between RBD and ACE2 to identify distribution for electrostatic complementarity and hydrophobic contact within interphase [17]. Piplani et al. studied the binding affinity of RBD with ACE2 in various species, including humans, to describe higher infectivity through interaction energy comparison in humans [11].

On the other hand, S proteins are frequently variable, leading to variation in the neutralizing antibodies produced by the host [18]. From April 2020, the D614G mutation of the S protein exhibited major variation, and D614G has since become the dominant mutant form of the S protein in the pandemic [19]. Most research into the S protein has focused on the RBD in theoretical computations [20,21,22] or biological assays [23,24,25]. However, whole-structure-based research is essential for analyzing conformational changes. Mutations such as D614G cannot be explained using only research into RBDs, as D614G is positioned at the CT2 domain, not at RBD. The ACE2 interaction process affects the whole structure of the S protein by causing conformational changes [26]. The Korea Centers for Disease Control and Prevention (KDCA) investigated the genomes of Korean patients and identified multiple mutation types in the S protein. Variants in the S protein, such as D614G, D614A, L455F, F456L, and Q787H, have been identified. The D614 residue is located in the CT2 domain of the S1 region, as before-mentioned, and the L455F and F456L residues are in the RBD region of the S1 domain. The Q787 residue is in the S2 region, not in the S1, and in the post-fusion state, is cleaved off by furin. In this study, considering the mutant types observed by KDCA, we used the whole structures, including both S protein and ACE2, for computational analysis using molecular dynamics (MD) simulations. Until now, whole structure-based analysis has not been studied in depth. ACE2–whole spike complex structures, including the RBD domain, were recently released [8] and were chosen for use in analyzing the structural differences caused by different mutation types. This research into mutation types involving the whole S protein–ACE2 complex provides insights into different biological phenomena in viral variants.

## 2. Results

### 2.1. SARS-CoV-2 S Protein Genome/Protein Analysis in Patients with COVID-19

We identified five non-synonymous mutations (L455F, F456L, D614G, D614A, and Q787H) that could affect the structure of the SARS-CoV-2 S protein, based on analysis of whole-genome sequence from COVID-19 patients in South Korea. The L455F and F456L mutation of the S protein were located in receptor-binding domain associated with ACE2 interaction of host cell. The D614G and D614A mutations of the S protein were identified at the same location as amino acids classified G, GH, GR, and GV clade. The Q787H mutation of the S protein was located at cleavage site of the S2 subunit protein.

### 2.2. Distance Analysis of V503 and N501 Residues among Trimeric S Protein Protomers

**Figure 1:**
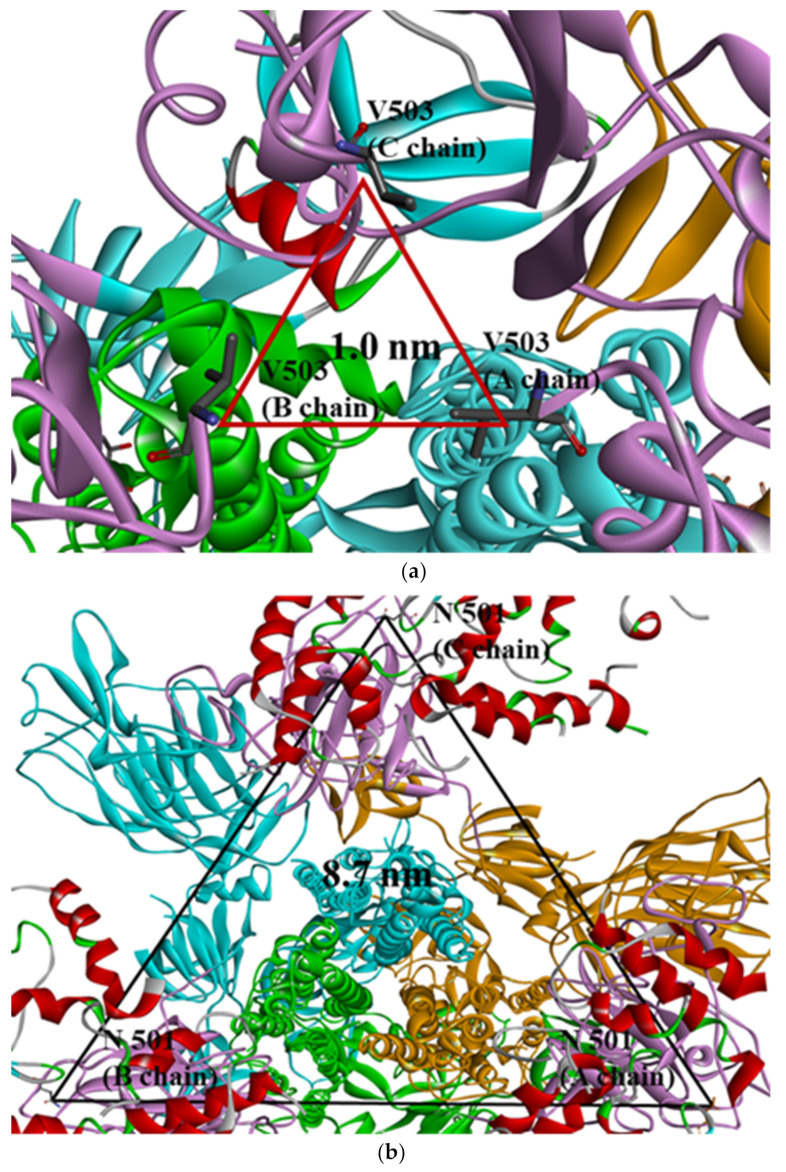
(**a**) Selection of quantifying V503 residues (PDB ID 6VXX) and (**b**) N501 residues (PDB ID 7A98) in the S protein. Trimeric RBDs in each chain are indicated in pink and ACE2 in red. Yellow, blue, and green colors indicate other domains except for RBD in each chain of S protein (**a**) V503 residues form threefold symmetry with the closest distance in the closed form (about 1.0 nm). (**b**) N501 residues form threefold symmetry with the same distance among chains (about 8.7 nm) and are involved in interphase interacting with ACE2 in the 3-open-complex form.

In this study, PDB data for SARS-CoV-2 S proteins complexed with ACE2 were used. In previous research, many studies focused only on the interactions between the RBD and ACE2 or the whole S protein without ACE2. This research, however, concentrated on whole trimeric structures with ACE2 in each open-complex form. MD simulation was conducted in vacuum with microcanonical (NVE) ensemble, an isolated system with particles (N) and energy (E) in a volume (V), due to SARSCoV-2 affecting respiratory systems such as influenza virus [27]. The root mean square deviation (RMSD) for Cα showed about 1 nm for whole residues and less than 0.2 nm for each domain in a chain of S protein regardless of mutant types (Table S1). These results were consistent with RMSD using whole residues within a chain in other research groups [28]. In order to identify conformational relationships among chains, V503 residues were confirmed as having the closest distance among the RBDs of chains in closed form (PDB ID 6VXX), with trimeric symmetry (Figure 1a) in whole trimeric S proteins. This quantification for V503 residues indicated that the RBD conformation among chains was stable when whole trimeric domains were considered. N501 residues were confirmed to have three symmetry distances, commonly about 8.7 nm in each of the three chains (Figure 1b), and were involved in ACE2-binding interphase in full open-complex form (PDB ID 7A98). The distance and standard deviation (SD) calculated among V503 and among N501 residues of chains in each of the 1-, 2-, and 3-open-complex forms fluctuated between S protein chains. These analytical methods were used to quantify the stability between the chains of whole structures caused by the V503 residue with three symmetries in closed form and the N501 residue with three symmetries considering the ACE2 interphase.

**Figure 2:**
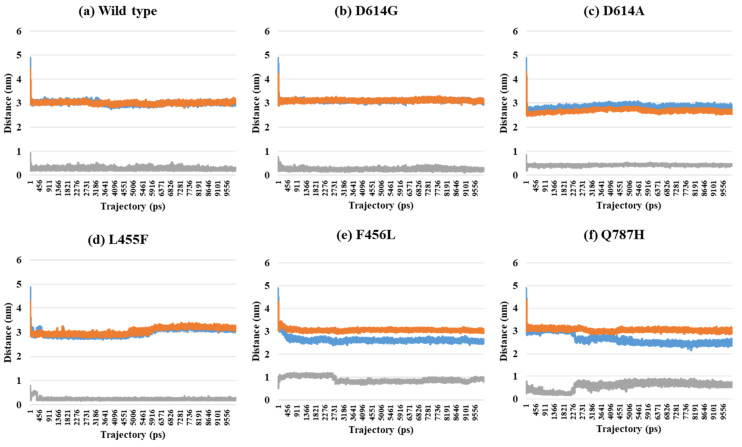
Distance between V503 residues in two chains depending on MD trajectories in the 1-open-complex form (7A94). (**a**) Wild type, (**b**) D614G, (**c**) D614A, (**d**) L455F, (**e**) F456L, and (**f**) Q787H. The X axis denotes trajectory (ps), and the Y axis denotes distance (nm). Blue color indicates the distance between A and B, orange indicates A–C distance, and gray indicates distance between B and C.

**Figure 3:**
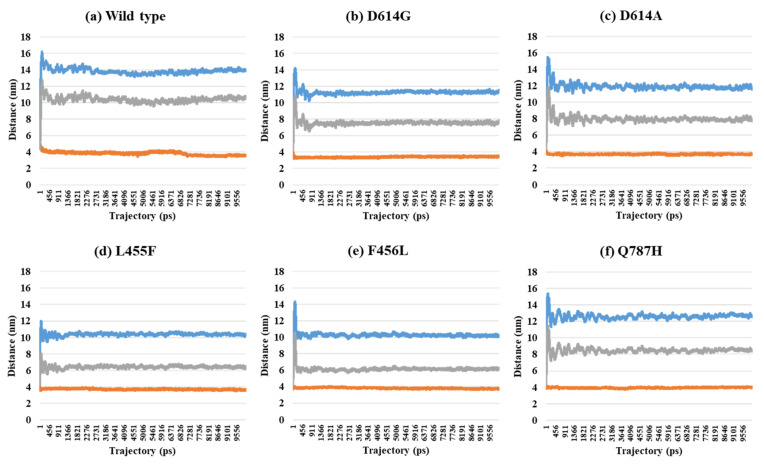
Distance between V503 residues in two chains depending on MD trajectories in the 2-open-complex form (7A97). (**a**) Wild type, (**b**) D614G, (**c**) D614A, (**d**) L455F, (**e**) F456L, and (**f**) Q787H. The X axis denotes trajectory (ps), and the Y axis denotes distance (nm). Blue color indicates the distance between A and B, orange indicates A–C distance, and gray indicates distance between B and C.

**Figure 4:**
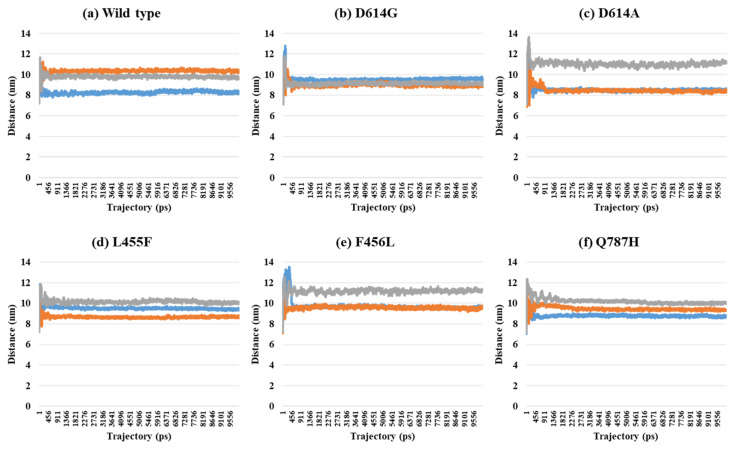
Distance between V503 residues in two chains depending on MD trajectories in the 3-open-complex form (7A98). (**a**) Wild type, (**b**) D614G, (**c**) D614A, (**d**) L455F, (**e**) F456L, and (**f**) Q787H. The X axis denotes trajectory (ps), and the Y axis denotes distance (nm). Blue color indicates the distance between A and B, orange indicates A–C distance, and gray indicates distance between B and C.

Depending on the MD trajectories, the distances of V503 and N501 residues among the three chains bound to ACE2 were analyzed in all open-complex forms (1-, 2-, and 3-open). In the 1-open-complex form, distance calculations showed no difference caused by different mutation types (Figure 2 and Table 1). The SD values for the distances in each chain remained below 0.5 nm. In the 2-open-complex form, all mutant types—D614G, D614A, L455F, F456L, and Q787H—showed lower distance and SD values than wild type (Figure 3 and Table 1). The SD values among chains were about 5 nm in the wild type and about 4 nm in mutants. In the 3-open-complex form, mutant types D614A and Q787H had lower SD values than the wild type, and D614G had the lowest SD value among the chains (Figure 4 and Table 1). The D614G mutant had a lower SD value of distance than the wild type in each complex form.

The distance fluctuations between N501 residues showed a pattern similar to those of V503 residues (Figures S1–S3 and Table S2). In the 1-open-complex form, there were no differences caused by different mutant types. The SD values for distances among each chain were below 0.5 nm. In the 2-open-complex form, the SD values among chains was about 4.5 nm in the wild type and about 4 nm in the mutant types D614G, D614A, L455F, and F456L. In the 3-open-complex form, D614G had the lowest SD value of 0.27 nm, similar to the values for the V503 residue. The quantification between the RBDs of the whole S protein indicated that the mutant types were more stable than the wild type. From the results for each open-complex form, the D614G mutant appeared to be more stable than the other mutant types in all open-complex forms. A summary of the quantification of the V503 residue distance is presented in Table 1 (V503) and that of N501 residue in Table S2 (N501).

### 2.3. Binding Free Energy Analysis through MM/PBSA Calculations between the S Protein and ACE2

**Figure 5:**
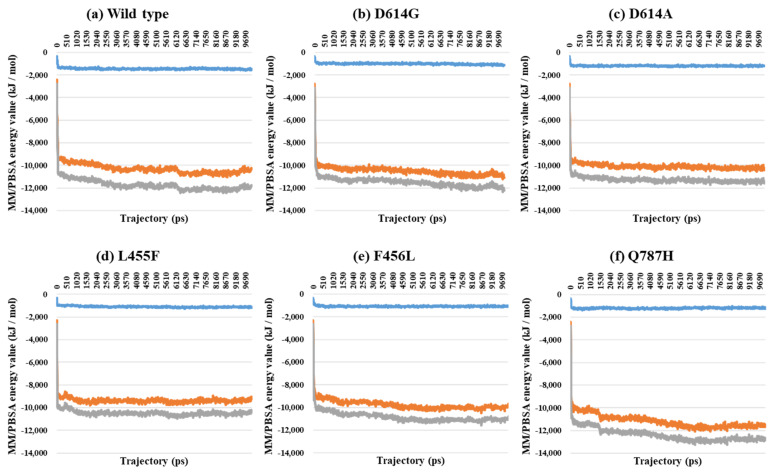
MM/PBSA result depending on the MD trajectories in the 1-open-complex form (7A94). (**a**) Wild type, (**b**) D614G, (**c**) D614A, (**d**) L455F, (**e**) F456L, and (**f**) Q787H. The X axis denotes trajectory (ps), and the Y axis denotes MM/PBSA energy value (kJ/mol). Blue color indicates van der Waals energy between S protein and ACE2, orange color indicates electrostatic energy, and gray color indicates total energy.

**Figure 6:**
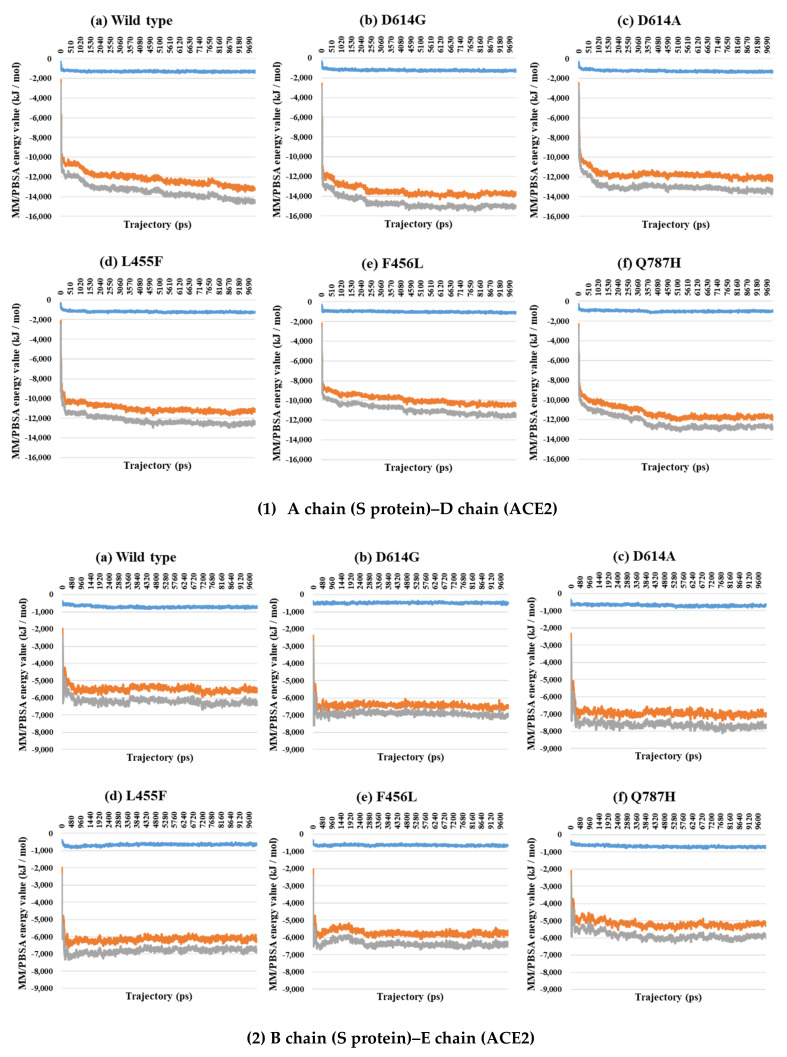
MM/PBSA results for A–D (1) and B–E (2) depending on the MD trajectories in the 2-open-complex form (7A97). (**a**) Wild type, (**b**) D614G, (**c**) D614A, (**d**) L455F, (**e**) F456L, and (**f**) Q787H. The X axis denotes trajectory (ps), and the Y axis denotes the MM/PBSA energy value (kJ/mol). Blue color indicates van der Waals energy between S protein and ACE2, orange color indicates electrostatic energy, and gray color indicates total energy.

**Figure 7:**
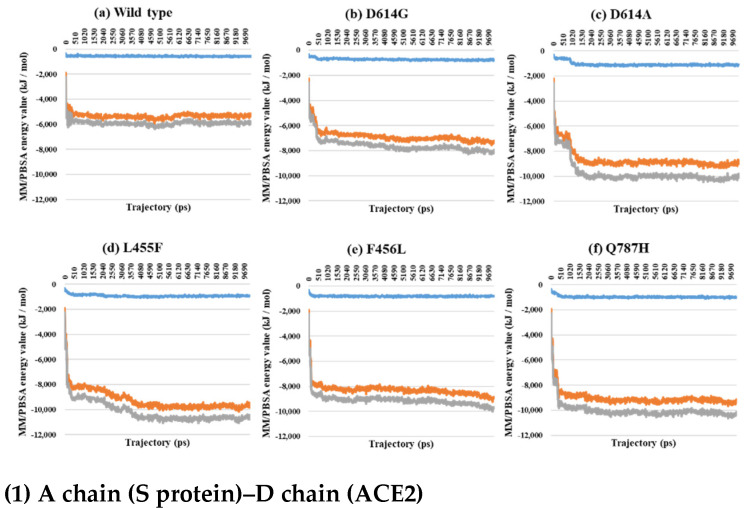

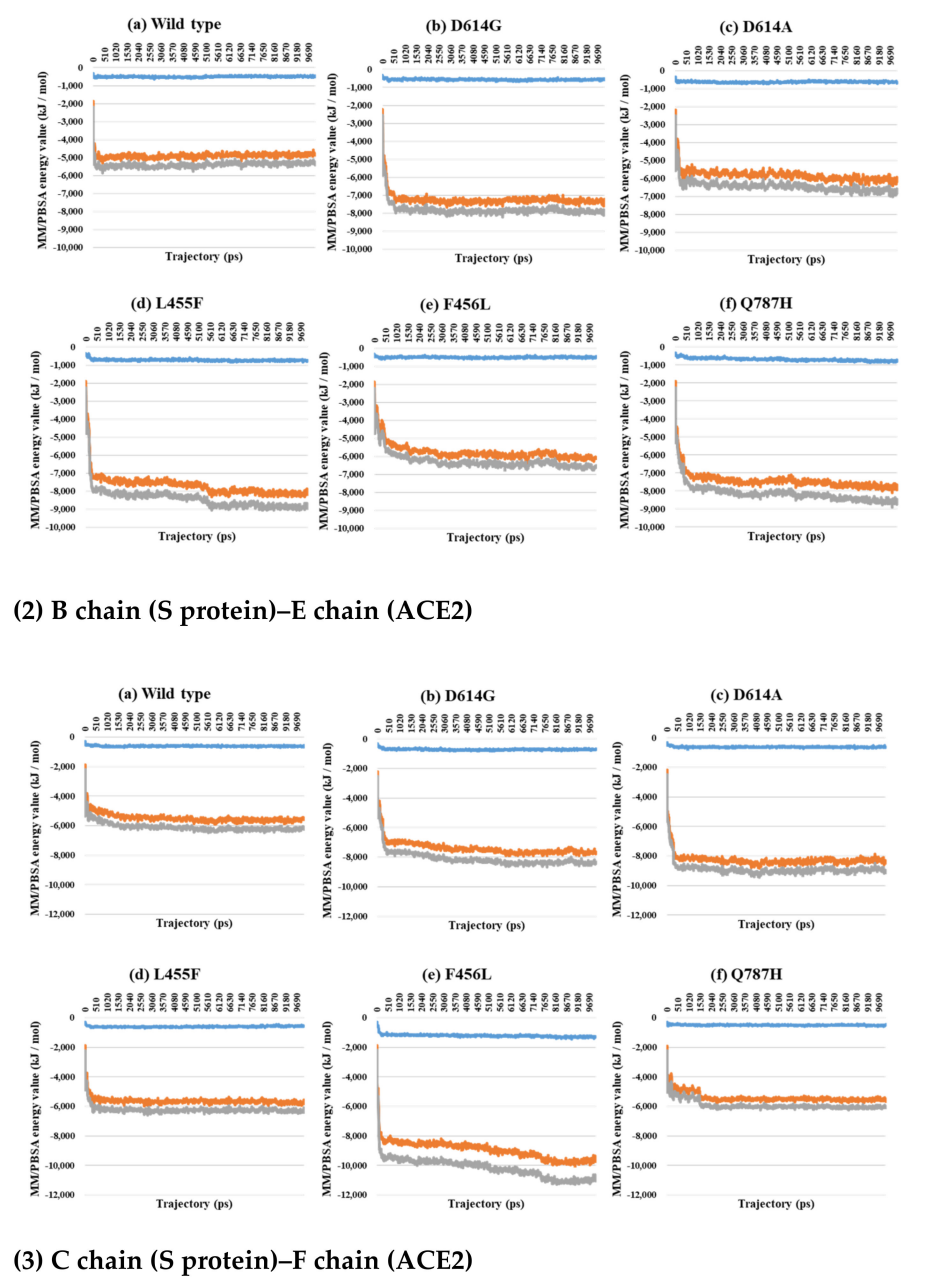
MM/PBSA for A–D (1), B–E (2), and C–F (3) depending on the MD trajectories in the 3-open-complex form (7A98). (**a**) Wild type, (**b**) D614G, (**c**) D614A, (**d**) L455F, (**e**) F456L, and (**f**) Q787H. The X axis denotes trajectory (ps), and the Y axis denotes MM/PBSA energy value (kJ/mol). Blue color indicates van der Waals energy between S protein and ACE2, orange color indicates electrostatic energy, and gray color indicates total energy.

Almost all interaction energies for all RBD–ACE2 complex structures have previously been studied. Our research was focused on the energy calculation for all atoms between the full-length S protein and ACE2 using MM/PBSA calculation. The MM/PBSA values were presented as van der Waals energy, electrostatic energy, and total energy. In the 1-open-complex form, the D614G and Q787H mutants had lower MM/PBSA values (Figure 5 and Table 2). In the 2-open-complex form, MM/PBSA was calculated between the S protein and ACE2 for the A–D and B–E chains in two sets. The D614G mutant had the lowest stable energy (Figure 6 and Table 3). Mutants D614A, L455F, F456L, and Q787H had energy values no lower than the wild type. The D614G mutant remained stable in both steps. In the 3-open-complex form, MM/PBSA was calculated for the S protein and ACE2 for the A–D, B–E, and C–F chains in three sets. The wild type had the highest energy between the S protein and ACE2 compared to all of the mutants. D614G was also a stable mutant (Figure 7 and Table 4). In the 2-open-complex form, the RBD mutants L455F and F456L were more unstable than the wild type. In the 1-open-complex form, the Q787H mutant in the S2 region had lower energy values but not in the 2-open-complex form. D614G had lower energy than the wild type in all steps.

## 3. Discussion

SARS-CoV-2 is an RNA virus causing severe pandemic disease. The S protein in SARS-CoV-2 is frequently mutated. KDCA investigated S protein variants of SARS-CoV-2 in Korean patients using RT-PCR analysis and identified five major mutants (D614G, D614A, L455F, F456L, and Q787H) depending on domain region. In general, it was well-known that S protein interacts with human ACE2 and penetrates the human cell. RBD of all domains in S protein is a direct interaction domain with ACE2. Therefore, much research into the S protein of the virus has focused on the RBD in mutation-related researches. Francesco and colleagues investigated the differences in interactions between the RBD and ACE2 depending on RBD mutant types using the GBPM score in multiple co-crystal structures [29]. Other research groups have studied differences in interactions in the RBD–ACE2 dynamic environment using MD simulation [10,14]. Many advanced structural studies have been conducted addressing the interactions between the RBD and ACE2. The whole structure, however, is needed for detailed analysis because the S protein consists of a whole trimeric structure in its usual environment, and mutations in other regions, as well as RBD domain mutants, could affect the characteristics of the system and hence the course of the pandemic. In an investigation into Korean mutants obtained from the KDCA, the D614G mutant was confirmed as the most prevalent, as it is in other countries. The D614 residue is located in the CT2 domain, not in the RBD. The D614G mutant has both distance and energy stability, according to the results of the V503 and N501 residue distance and MM/PBSA energy calculations. The D614G mutant had lower distance fluctuations between the S protein chains, indicating the presence of protomers, and lower energy between the S protein and ACE2 in the 1-, 2-, and 3-complex forms than the wild type. L455F and F456L are located in the RBD region, which interacts with ACE2. Computational studies using only RBD structure showed binding energy stability for RBD mutants other than wild type but were not actively studied for the whole structure [30]. The Q787H region is in the S2 region and is cleaved by furin post fusion. The other mutants—D614A, L455F, F456L, and Q787H—show more stability than the wild type in some complexes and are more unstable than the wild type in other situations. The results of the calculation of energy between whole residues in the S protein and ACE2 indicate the importance of conformation due to the presence of regions other than the RBD. The RBD of the S protein is involved in direct binding with ACE2, but the physicochemical properties, including those of whole domains, should be considered when investigating conformation. Interactions between the 614 residue and a residue in the adjacent chain arise from hydrogen bonding [31,32]. The shortening of the amino acid chain by a D614 mutation affects the interaction among the S proteins, causing them to be more flexible. D614G is missing a side chain, which may affect flexibility more than D614A. Leonid et al. investigated the conformational relationship using ACE2-free whole S protein using electron microscopy. The results showed that the D614G mutation affected no neutralization by antibody but led to a more frequent transition from closed to open form than is observed in the wild type, causing higher viral infectivity [26]. Rachel et al. compared the number of residues within interphase for the wild type and the D614G mutant using each closed-form and 1-open form without ACE2 [33]. The number of residues in the D614G mutant was higher in the open form than in the wild type, although there was no difference in the closed form. Previous studies into the protein conformation used ACE2-free whole S protein, lacking the structure of the complex, and those results suggest that the importance of conformation depends on the mutant type. Other computational methods were used in our research because we could use the recently released structure of the whole S protein/ACE2 complex. Our results suggest that the conformational importance of the D614G mutant in the CT2 domain was related more to the interactions between S protein chains than to the effects of other mutants. The large prevalence of D614G is in Korea, and this could affect the worldwide pandemic. We suggest targeting conformational regions such as the 614 residue for therapy rather than directly targeting the RBD-binding ACE2.

## 4. Materials and Methods

### 4.1. Ethical Considerations

This study has been approved by the Institutional Review Board at the Korea Centers for Disease Control and Prevention (2020-03-01-P-A) and is considered to be a public health act to the outbreak. Thus, the board has waived the requirement for written consent as outlined in the Title Laboratory Respondence to COVID-19. All the methods presented in this study were conducted in accordance with the relevant guidelines and regulations.

### 4.2. Genome/Protein Sequence Investigation of Korean Patients with COVID-19

Nasopharyngeal and oropharyngeal swab specimens were collected from symptomatic patients to detect severe acute respiratory syndrome coronavirus 2 (SARS-CoV-2) by real-time reverse transcriptase-polymerase chain reaction (RT-PCR). RNA was extracted from the specimens using a Qiagen viral RNA mini kit (Qiagen, Hilden, Germany) according to the manufacturer’s protocol. Next, real-time RT-PCR was performed on the cycle threshold value of the SARS-CoV-2 target gene (ORF 1b and E) [34]. For whole-genome sequencing, cDNA was amplified using the ARTIC primer pools (https://artic.network/ncov-2019v3, accessed on 24 March 2020). DNA libraries were extracted using the Nextera DNA Flex Library Prep Kit (Illumina, San Diego, CA, USA), and sequencing was performed on the MiSeq instrument using a MiSeq reagent kit V2 (Illumina, San Diego, CA, USA) to obtain an average genome coverage greater than 1000× for all the isolates. The reads were trimmed and mapped to reference genome MN908947.3 using CLC Genomics Workbench version 20.0.3 (CLC Bio, Aarhus, Denmark). Using the reference genome, single-nucleotide variants (SNVs) were called using the BioNumerics version 7.6 SARS-CoV-2 plugin (Applied Maths, Sint-Martens-Latem, Belgium) [35].

### 4.3. Dataset Preparation

The 3D structures of full-length SARS-CoV-2 S protein complexed with ACE2 were selected prior to the analysis. PDB structures 7A94, 7A97, and 7A98 were selected as examples of 1-,2-, and 3-open complexes with ACE2 [8]. Point mutations investigated in Korean patients with COVID-19 were applied to D614, L455, F456, and Q787 residues using Discovery Studio 2020 (BIOVIA, San Diego, CA, USA). Overall, 18 structures (the 1-, 2-, and 3-open-complex forms for the wild type, D614G, D614A, L455F, F456L, and Q787H) were built as the inputs to the MD simulation calculation.

### 4.4. MD Simulation

All MD simulations were performed using the GROMACS 5.1.3 package [36] with the CHARMM27 force field [37]. The PDB entries 7A94, 7A97, and 7A98 [8] were used as the starting point for the simulations. MD simulations were performed at 298 K in a cubic box with molecules. The system was minimized using 50,000 steps of the steepest descent algorithm to ensure that the system had no steric clashes, inappropriate geometry, or structural distortions. MD production was conducted under NVE ensembles in a vacuum phase [27]. The Linear Constraint Solver algorithm was used to constrain bond lengths. Long-range electrostatic interactions were treated using the particle-mesh Ewald summation method [38], and a cutoff of 10 Å was used for short-range interactions. Production runs were performed for 10 ns [39]. The integration time-step was set to 1 fs, and the trajectory coordinates and energies were saved at 1 ps intervals. The built-in programs of the GROMACS software package were used for the analysis.

### 4.5. V503 and N501 Residue Distance Calculation

V503 and N501 residues in trimeric S protein chains were indexed using the “gmx index” module. The distance between V503 residues and between N501 residues among A–B, A–C, and B–C chains were calculated as the minimum distance in the “gmx mindist” module. Standard deviations (SDs) between V503 residues and between N501 residues in two chains and among three chains, such as A–B and A–C, A–B and B–C, A–C and B–C, and A–B–C, were calculated from distance values depending on trajectories. The unit for distance was nanometers (nm).

### 4.6. Binding Free Energy Analysis

In order to calculate the binding free energy of S protein–ACE2 complexes, the molecular mechanics/Poisson–Boltzmann surface area (MM/PBSA) calculation [40] was conducted using “g_mmpbsa” in MD trajectory files. All atoms’ interaction energies between the whole domains of the spike, not only the RBD, and ACE2 were calculated. The structure of 7A94 (1-open-complex form) was used for MM/PBSA calculation between the A–D chains, and the structure of 7A97 (2-open-complex form) was used for MM/PBSA calculation between the A–D and B–E chains. The structure of 7A98 (3-open-complex form) was used for MM/PBSA calculation between the A–D, B–E, and C–F chains. Because of the long computation time, MM/PBSA was calculated at 10 ps intervals. The van der Waals, electrostatic, and total energies were represented as kJ/mol in the vacuum phase.

## Figures and Tables

**Table 1:** Summary of distance and SD between V503 residues (nm). The values were measured in the final trajectory (10 ns).

PDB	Mutant Type	A–B	A–C	B–C	SD (A–B) and (A–C)	SD (A–B) and (B–C)	SD (A–C) and (B–C)	SD (A–B–C)
7A94 (1-open-complex form)	Wild	3.00	3.01	0.25	0.01	1.95	1.95	1.59
	D614G	3.05	2.99	0.27	0.04	1.97	1.93	1.59
	D614A	2.82	2.65	0.44	0.12	1.68	1.56	1.33
	L455F	3.13	3.23	0.25	0.07	2.04	2.11	1.69
	F456L	2.53	3.01	0.85	0.34	1.19	1.53	1.14
	Q787H	2.47	2.95	0.59	0.34	1.33	1.67	1.25
7A97 (2-open-complex form)	Wild	13.96	3.57	10.63	7.35	2.36	5.00	5.31
	D614G	11.55	3.47	7.79	5.71	2.66	3.05	4.04
	D614A	11.65	3.72	7.72	5.61	2.78	2.83	3.97
	L455F	10.26	3.60	6.48	4.71	2.67	2.04	3.34
	F456L	10.30	3.79	6.20	4.60	2.90	1.70	3.29
	Q787H	12.55	4.03	8.38	6.03	2.95	3.08	4.26
7A98 (3-open-complex form)	Wild	8.23	10.32	9.75	1.48	1.08	0.40	1.08
	D614G	9.61	8.81	9.14	0.56	0.33	0.23	0.40
	D614A	8.63	8.51	11.18	0.08	1.81	1.89	1.51
	L455F	9.33	8.65	10.05	0.48	0.51	0.99	0.70
	F456L	9.69	9.63	11.30	0.04	1.14	1.18	0.95
	Q787H	8.72	9.30	10.04	0.41	0.93	0.53	0.66

**Table 2:** Summary of MM/PBSA between the S protein and ACE2 in the 1-open-complex form. VdW indicates van der Waals energy, E denotes electrostatic energy, and T is total energy (kJ/mol). The values were measured in the final trajectory (10 ns).

PDB	Mutant Type	VdW	E	T
7A94	Wild	−1461.5	−10,311.8	−11,773.3
	D614G	−1129.2	−11,082.0	−12,211.2
	D614A	−1186.4	−10,417.5	−11,603.9
	L455F	−1185.4	−9224.2	−10,409.6
	F456L	−1120.6	−9704.0	−10,824.6
	Q787H	−1236.5	−11,629.0	−12,865.5

**Table 3:** Summary of MM/PBSA between the S protein and ACE2 in the 2-open-complex form. VdW indicates van der Waals energy, E denotes electrostatic energy, and T is total energy (kJ/mol). The values were measured in the final trajectory (10 ns).

PDB	Mutant Type	VdW	E	T
7A97 A–D chain interaction	Wild	−1326.4	−13,200.4	−14,526.7
	D614G	−1290.0	−13,844.8	−15,134.7
	D614A	−1307.6	−12,053.5	−13,361.1
	L455F	−1237.6	−11,097.9	−12,335.5
	F456L	−1081.2	−10,342.6	−11,423.8
	Q787H	−948.2	−12,017.3	−12,965.5
7A97 B–E chain interaction	Wild	−677.3	−5553.0	−6230.3
	D614G	−483.8	−6481.6	−6965.5
	D614A	−674.9	−6940.9	−7615.8
	L455F	−664.7	−6134.4	−6799.1
	F456L	−646.3	−5697.0	−6343.3
	Q787H	−693.7	−5303.3	−5997.0
7A97 Total	Wild	−2003.7	−18,753.4	−20,757.0
	D614G	−1773.8	−20,326.4	−22,100.2
	D614A	−1982.5	−18,994.4	−20,976.9
	L455F	−1902.3	−17,232.3	−19,134.6
	F456L	−1727.4	−16,039.6	−17,767.1
	Q787H	−1641.9	−17,320.6	−18,962.5

**Table 4:** Summary of MM/PBSA between the S protein and ACE2 in the 3-open-complex form. VdW indicates van der Waals energy, E denotes electrostatic energy, and T is total energy (kJ/mol). The values were measured in the final trajectory (10 ns).

PDB	Type	VdW	E	T
7A98 A–D chain interaction	Wild	−535.4	−5278.5	−5813.8
	D614G	−735.3	−7198.1	−7933.4
	D614A	−1096.6	−9024.3	−10,120.8
	L455F	−946.6	−9730.1	−10,676.7
	F456L	−791.3	−8938.8	−9730.1
	Q787H	−973.9	−9216.2	−10,190.1
7A98 B–E chain interaction	Wild	−527.7	−4872.3	−5400.0
	D614G	−585.3	−7306.0	−7891.2
	D614A	−651.4	−6007.5	−6658.8
	L455F	−778.5	−8190.2	−8968.7
	F456L	−492.9	−6111.3	−6604.2
	Q787H	−759.2	−7916.7	−8675.9
7A98 C–F chain interaction	Wild	−583.0	−5587.4	−6170.4
	D614G	−715.8	−7689.6	−8405.4
	D614A	−638.3	−8485.6	−9124.0
	L455F	−582.9	−5819.0	−6401.9
	F456L	−1289.9	−9654.7	−10,944.6
	Q787H	−434.2	−5656.1	−6090.3
7A98 Total	Wild	−1646.1	−15,738.2	−17,384.2
	D614G	−2036.4	−22,193.7	−24,230.0
	D614A	−2386.3	−23,517.3	−25,903.6
	L455F	−2308.0	−23,739.2	−26,047.3
	F456L	−2574.0	−24,704.9	−27,278.9
	Q787H	−2167.3	−22,789.0	−24,956.3

## Data Availability

The SARS-CoV-2 whole-genome sequences described are available in GISAID (Accession IDs EPI_ISL_497968, 510610, 516783, 850199).

## Supplementary Materials

The following are available online at https://www.mdpi.com/article/10.3390/ijms22168714/s1, Figure S1. The distance between N501 residues in two chains depending on MD trajectories in 1-open-complex form (7A94). Figure S2. The distance between N501 residues in two chains depending on MD trajectories in 2-open-complex form (7A97). Figure S3. The distance between N501 residues in two chains depending on MD trajectories in 3-open-complex form (7A98). Table S1. The RMSD value within a chain of S protein (nm). Table S2. Summary for distance and S.D between V503 residues (nm).
