# Supplementary material for: Molecular Dynamics Studies on the Structural Characteristics for the Stability Prediction of SARS-CoV-2
Source: Int J Mol Sci. 2021 Aug 13;22(16):8714. doi: 10.3390/ijms22168714 (PMC8395978; doi:10.3390/ijms22168714)
Supplement: Supplementary file 1 [file ijms-22-08714-s001.zip › ijms-1328396-supplementary.pdf]

Supplementary Information

Molecular Dynamics Studies on the structural characteristics for the stability prediction of SARS-CoV-2

Kwang-Eun Choi[1†], Jeong-Min Kim[2†], JeeEun Rhee[2], Ae Kyung Park[2], Eun-Jin Kim[2] and Nam Sook Kang [1*]

[1]*Graduate School of New Drug Discovery and Development, Chungnam National University, Daejeon 305-764, Republic of Korea*

[2]*Division of Emerging Infectious Diseases, Bureau of Infectious Disease Diagnosis Control, Korea Disease Control and Prevention Agency, 187 Osongsaengmyeong 2-ro, Osong-eup, Heungdeok-gu, Cheongju-si, Chungcheongbuk-do 28159, Republic of Korea*

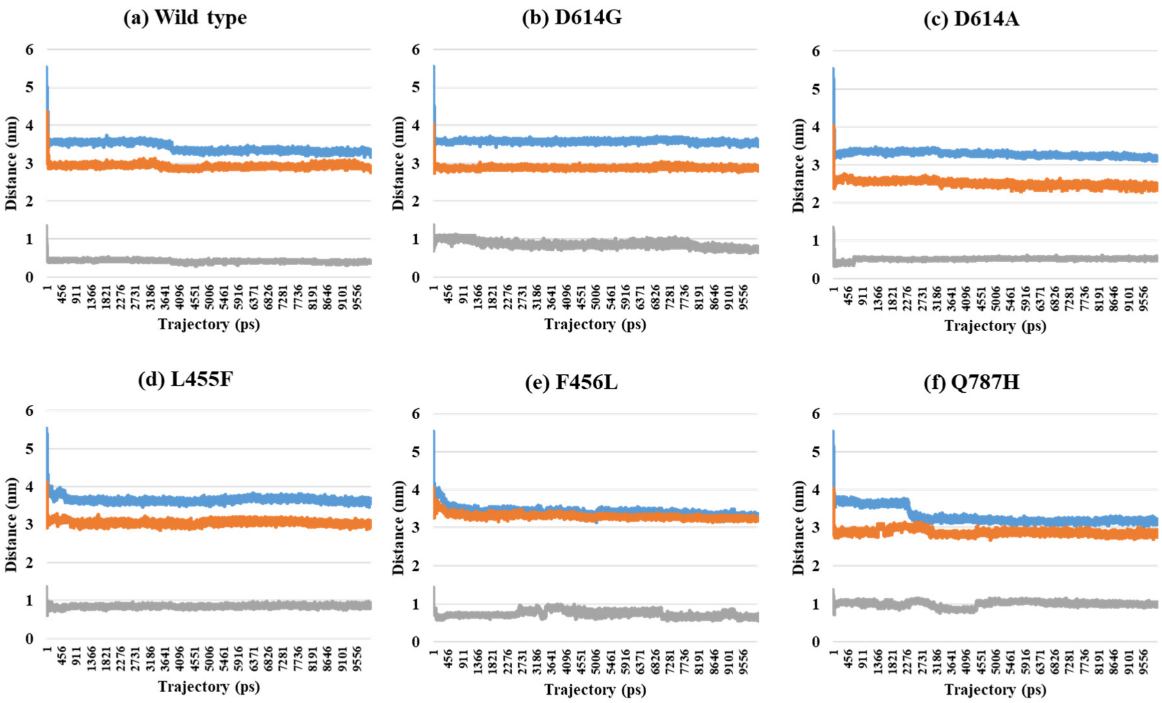

Figure S1. The distance between N501 residues in two chains depending on MD trajectories in 1-open complex form (7A94). (a) Wild type, (b) D614G, (c) D614A, (d) L455F, (e) F456L, and (f) Q787H. The X axis denotes trajectory (ps), and the Y axis denotes distance (nm). Blue color indicates the distance between A-B, orange indicates A-C distance, and gray in-dicates distance between B-C.

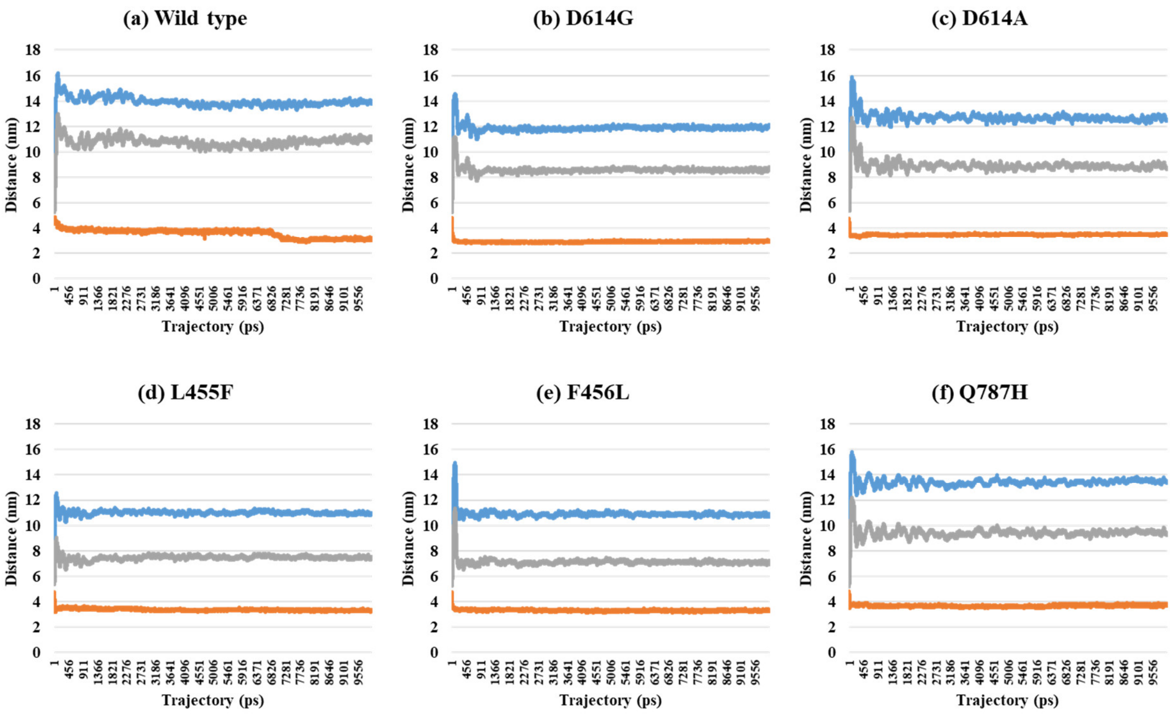

Figure S2. The distance between N501 residues in two chains depending on MD trajectories in 2-open complex form (7A97). (a) Wild type, (b) D614G, (c) D614A, (d) L455F, (e) F456L, and (f) Q787H. The X axis denotes trajectory (ps), and the Y axis denotes distance (nm). Blue color indicates the distance between A-B, orange indicates A-C distance, and gray in-dicates distance between B-C.

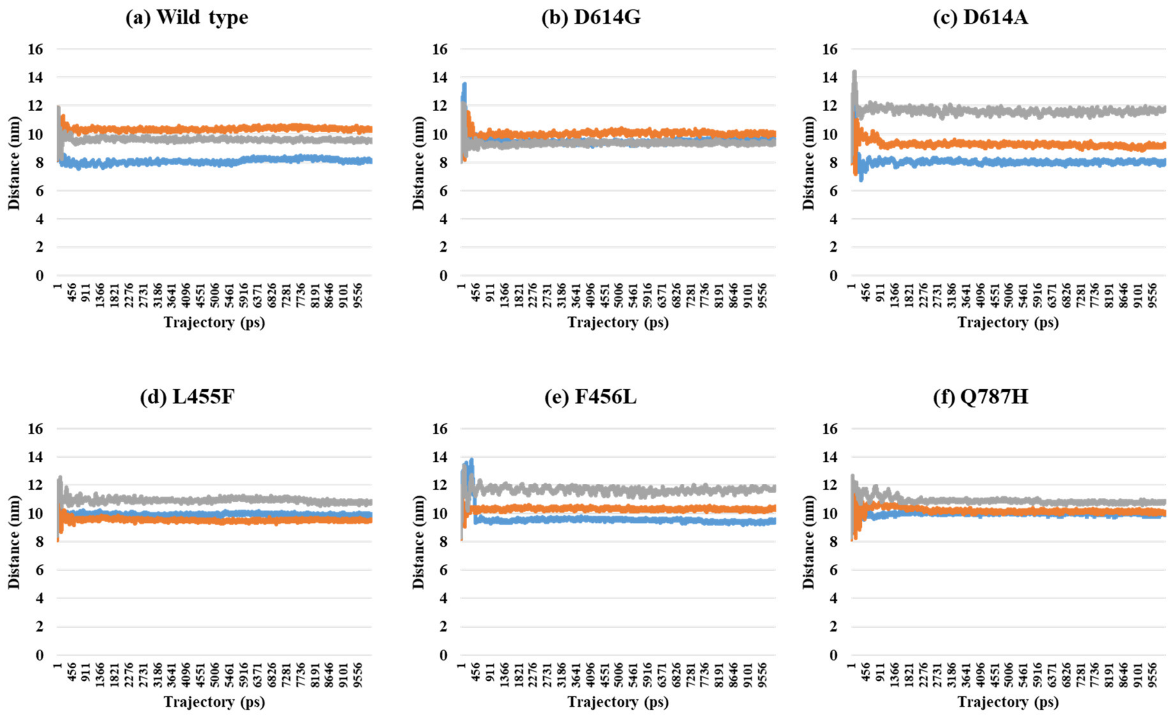

Figure S3. The distance between N501 residues in two chains depending on MD trajectories in 3-open complex form (7A98). (a) Wild type, (b) D614G, (c) D614A, (d) L455F, (e) F456L, and (f) Q787H. The X axis denotes trajectory (ps), and the Y axis denotes distance (nm). Blue color indicates the distance between A-B, orange indicates A-C distance, and gray in-dicates distance between B-C.

Table S1. The RMSD value within a chain of S protein (nm). The calculation values were presented in final trajectory (10 ns).

Residue	Wild	D614G	D614A	L455F	F456L	Q787H
**Whole**	1.03	1.05	0.98	1.05	1.05	1.10
**RBD**	0.44	0.30	0.40	0.40	0.52	0.32
**Other domains**	0.79	0.81	0.73	0.88	0.82	0.90

Table S2. Summary for distance and S.D between V503 residues (nm). The calculation values were presented in final trajectory (10 ns).

PDB	Mutant type	A-B	A-C	B-C	SD (A-B) & (A-C)	SD (A-B) & (B-C)	SD (A-C) & (B-C)	SD (A-B-C)
**7A94 (1-open complex form)**	**Wild**	3.33	2.91	0.43	0.30	2.05	1.75	1.57
	**D614G**	3.54	2.86	0.68	0.48	2.02	1.54	1.49
	**D614A**	3.22	2.47	0.52	0.53	1.91	1.38	1.39
	**L455F**	3.65	3.04	0.89	0.43	1.95	1.52	1.45
	**F456L**	3.28	3.21	0.66	0.05	1.85	1.80	1.49
	**Q787H**	3.18	2.76	0.95	0.30	1.57	1.28	1.18
**7A97 (2-open complex form)**	**Wild**	13.93	3.10	11.08	7.66	2.02	5.64	4.66
	**D614G**	12.15	2.93	8.80	6.52	2.37	4.15	3.88
	**D614A**	12.47	3.51	8.70	6.34	2.67	3.67	3.80
	**L455F**	10.89	3.27	7.49	5.39	2.40	2.99	3.25
	**F456L**	10.92	3.34	7.15	5.36	2.67	2.69	3.22
	**Q787H**	13.43	3.81	9.29	6.80	2.93	3.87	4.07
**7A98 (3-open complex form)**	**Wild**	8.04	10.29	9.56	1.59	1.07	0.52	1.15
	**D614G**	9.60	9.94	9.41	0.24	0.14	0.37	0.27
	**D614A**	8.02	9.24	11.71	0.87	2.61	1.75	1.88
	**L455F**	9.83	9.49	10.77	0.24	0.66	0.90	0.66
	**F456L**	9.48	10.34	11.82	0.60	1.65	1.05	1.18
	**Q787H**	9.98	10.02	10.77	0.03	0.56	0.53	0.45
